# Rapid Response of Eastern Mediterranean Deep Sea Microbial Communities to Oil

**DOI:** 10.1038/s41598-017-05958-x

**Published:** 2017-07-18

**Authors:** Jiang Liu, Stephen M. Techtmann, Hannah L. Woo, Daliang Ning, Julian L. Fortney, Terry C. Hazen

**Affiliations:** 1 Department of Microbiology, University of Tennessee Knoxville, Knoxville, TN USA; 2 Department of Biological Sciences, Michigan Technological University, Houghton, MI USA; 3 Department of Civil & Environmental Engineering, University of Tennessee, Knoxville, TN USA; 4 Department of Earth & Planetary Sciences, University of Tennessee, Knoxville, TN USA; 5 Consolidated Core Laboratory, Institute for Environmental Genomics and Department of Microbiology and Plant Biology, The University of Oklahoma, Norman, OK USA; 6 Biosciences Division, Oak Ridge National Lab, Oak Ridge, TN USA

## Abstract

Deep marine oil spills like the *Deepwater Horizon* (DWH) in the Gulf of Mexico have the potential to drastically impact marine systems. Crude oil contamination in marine systems remains a concern, especially for countries around the Mediterranean Sea with off shore oil production. The goal of this study was to investigate the response of indigenous microbial communities to crude oil in the deep Eastern Mediterranean Sea (E. Med.) water column and to minimize potential bias associated with storage and shifts in microbial community structure from sample storage. 16S rRNA amplicon sequencing was combined with *GeoChip* metagenomic analysis to monitor the microbial community changes to the crude oil and dispersant in on-ship microcosms set up immediately after water collection. After 3 days of incubation at 14 °C, the microbial communities from two different water depths: 824 m and 1210 m became dominated by well-known oil degrading bacteria. The archaeal population and the overall microbial community diversity drastically decreased. Similarly, *GeoChip* metagenomic analysis revealed a tremendous enrichment of genes related to oil biodegradation, which was consistent with the results from the DWH oil spill. These results highlight a rapid microbial adaption to oil contamination in the deep E. Med., and indicate strong oil biodegradation potential.

## Introduction

Offshore oil and gas prospecting and production continue to grow due to increasing demand worldwide. This in turn has increased the probability of oil spills in the oceans^1^. Crude oil can be a serious contaminant in the marine environment^2, 3^. Prior to the DWH oil spill of 2010 in the Gulf of Mexico (GOM), few studies investigated the impact of oil contamination in the deep ocean. During the DWH oil spill, oil was catastrophically released into the deep ocean underscoring the need to advance our understanding of oil biodegradation at both the ocean surface and in the bottom depths of the deep ocean^4^.

Since the American Petroleum Institute began to subsidize oil biodegradation research in 1942, more than 175 genera of microorganisms have been found with the capacity to utilize components from crude oil, making oil bioremediation a viable response strategy for oil spills^4, 5^. The environmental conditions of a marine habitat are known to impact both the identity of the oil degraders present as well as the rates of oil degradation. The physical and chemical properties of different locations in the oceans can vary greatly^6–9^. Therefore, the application of bioremediation methods must consider local conditions, the native microbial community, and how it varies from other well-characterized systems^10, 11^.

Large oil and gas reserves have recently been discovered in the E. Med. The E. Med. is a unique environment, in which the water column has high salinity and warm deepwater temperatures^6, 12, 13^. It is also known for very low nutrient concentrations compared to other deep ocean basins^14^. Because of these characteristics, the microbial community in the E. Med. may be distinct from other settings. For example, the E. Med. deep water microbial community may have a high abundance of mesophilic microbes^6, 12^.

During the DWH oil spill, different oil degrading bacteria were enriched at various stages within the deep-water oil plume^15^. *Oceanospirillales* dominated the microbial community in the early stages of the spill. Subsequently, *Colwellia* spp. as well as *Cycloclasticus* spp. became dominant in the water column. Many genes related to oil utilization were enriched immediately after the spill^16, 17^.

Also during the DWH oil spill, approximately 2.1 × 10^6^ gallons of dispersant (COREXIT 9500) was injected into the deep ocean for the first time to increase the hydrocarbon availability for deep marine oil degrading bacteria. In-lab studies show that dispersant can increase the biodegradation rate of crude oil^18, 19^. The availability of oil, nutrients, dispersant and other environmental factors are responsible for the succession of microbial communities^19–22^.

The E. Med. has great oil production potential and thus increasing deep sea oil spill risk, like the GOM. However, very limited information is available on E. Med. microbial community changes to oil contamination, especially in the deepwater column. The distinct geochemical and microbial features in E. Med. make it difficult to predict responses accurately based on GOM reports. Therefore, it is extremely important to investigate the deep ocean microbial response to crude oil. Our study aims to unveil the influence of crude oil and dispersant on the deep water microbial community in the E. Med. with limited storage effect. We employed a microcosm-based approach combined with 16S rRNA gene sequencing and *GeoChip* metagenomics analysis to characterize the microbial community response at two depths. 16S rRNA gene sequencing can provide very accurate *in situ* relative abundance and taxonomy information regarding the microbial community composition. *GeoChip* microarray measures functional genes and thus the *in situ* functional potential of environmental microbial communities with quite high sensitivity and in-depth details especially as it relates to oil and hydrocarbons^17, 19^.

As a novel aspect of this study, the microcosm experiments were set up on board the ship immediately after the collection of seawater. Performing on ship microcosms avoided long storage and shipping times, during which the microbial community structure can change drastically due to storage effects. Without storage effects, the findings of this microcosm study can be considered more representative of the natural *in situ* microbial community’s response to oil.

## Results

### Environmental features of the sampling site

**Figure 1:**
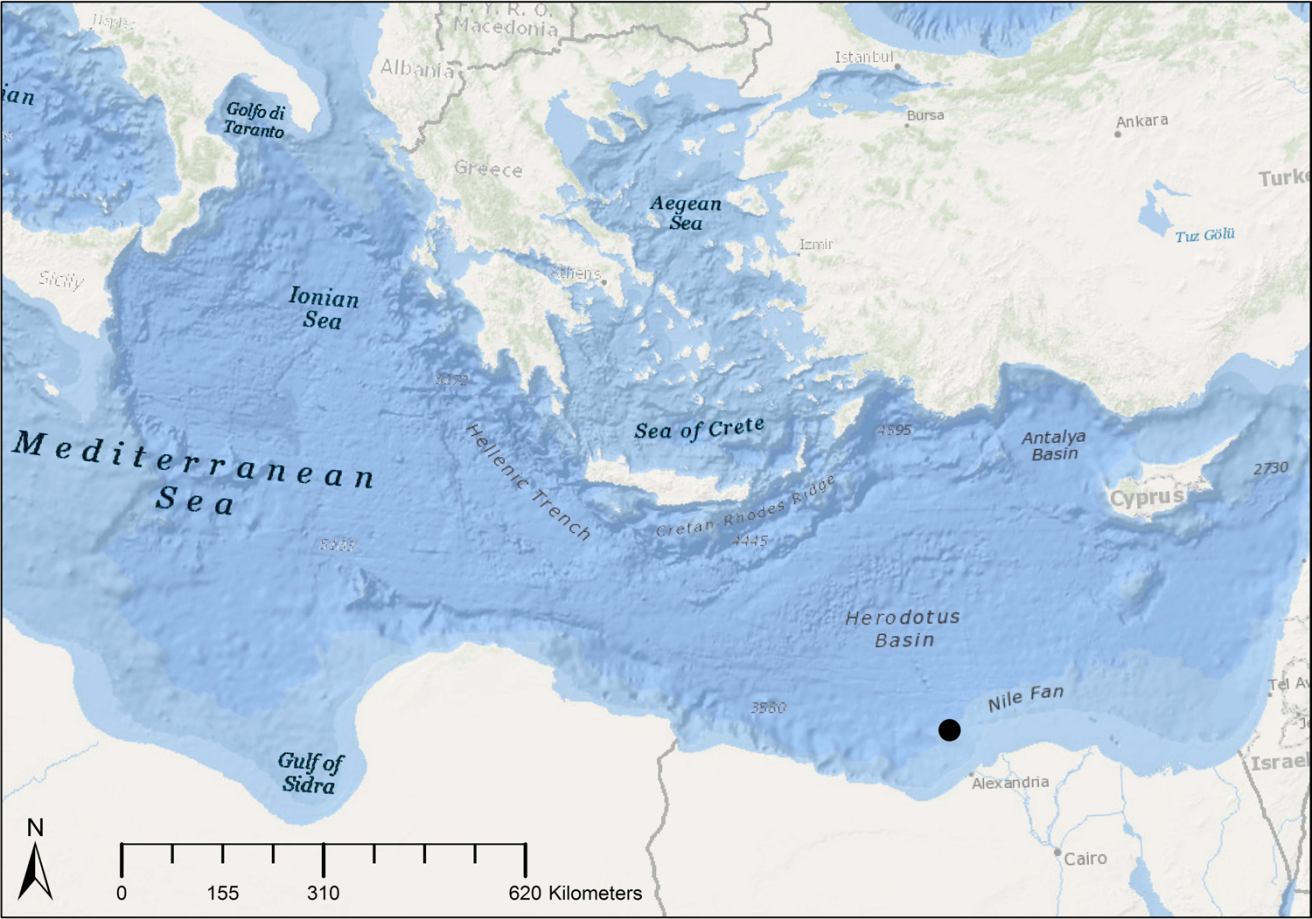
Map of the Sampling Site within E. Med. Sea. Map was created with ArcGIS. The samples were collected in the Nile Fan off the coast of Egypt.

During the BP E. Med., microbial survey cruise aboard the MV Fugro Navigator, seawater was collected at 834 and 1210 m deep, which are three-quarters and 50 m above the seabed at the sampling location, respectively (Fig. 1 and Table 1). The temperature, pH, and salinity of the samples were similar, but the dissolved oxygen was slightly higher at 1210 m. The E. Med. salinity of 38.8 practical salinity units (PSU) is slightly higher than the average ocean salinity^6^. The temperature at depth was 13.7 °C, which is much higher than other deep oceans such as the GOM^15^, which are typically around 4 °C.

**Table 1:** Sampling site physical and geochemical parameters.

	Latitude	Longitude	Depth (m)	Turbidity (FTU)	Temperature (°C)	Dissolved Oxygen (% Sat)	Pressure (db)	Salinity (psu)	pH
Sample 1	31.8058	29.5683	824	1.313	13.772	69.348	824.441	38.828	8.192
Sample 2	31.8058	29.5683	1210	1.313	13.788	65.432	1218.386	38.826	8.179

The samples were taken from the same site at different depths of the water column in 2012. Temperature, salinity, dissolved oxygen, pH and turbidity were measured using a CTD.

### Analysis of microbial diversity

#### Sequencing quality and microbial community structure

The 16S rRNA amplicon sequencing generated a total of 2,437,232 reads from 23 samples that were 250 base pairs long. The average number of sequences from each sample was 105,966. One sample, the 1210 m control at timepoint 1, did not produce enough sequencing reads and was excluded from all analysis.

**Figure 2:**
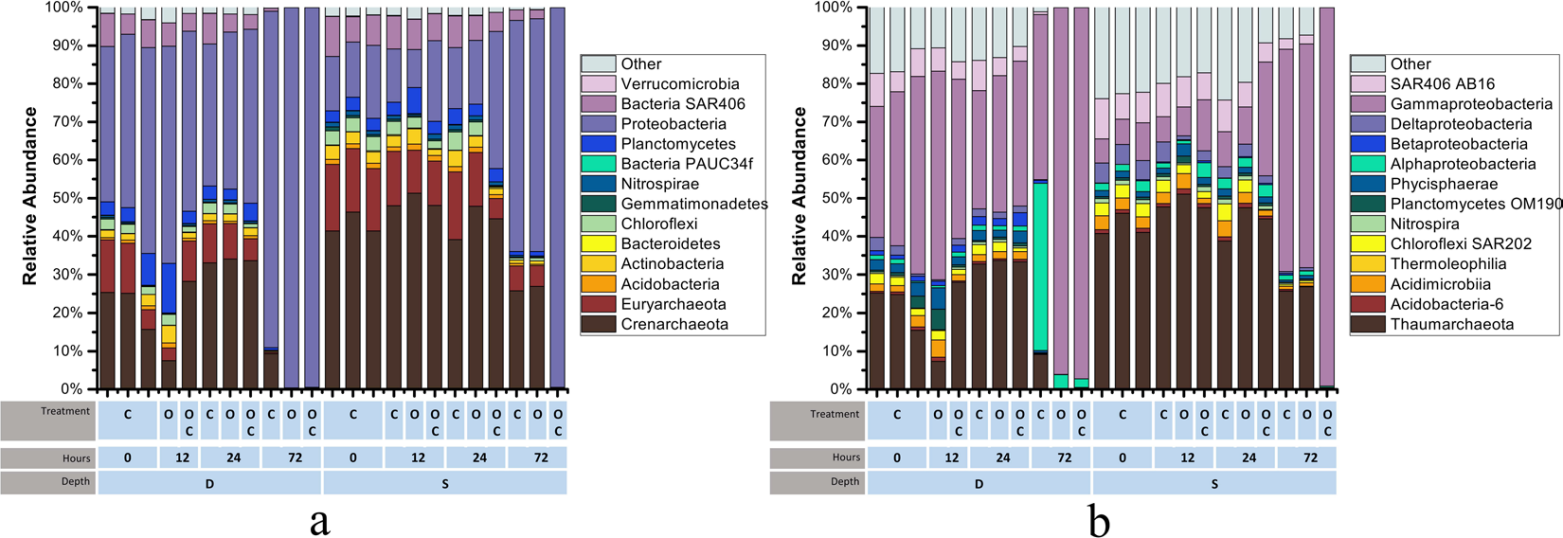
Taxa Plots of the Microbial community. (**a**) Total Microbial Community at the Phylum Level, (**b**) Total Microbial Community at the Class level. The samples are labeled with their treatment, timepoint and depth. C = control, O = oil-amendment, OC = oil with dispersant. Timepoints in h: 0 = initial, 1 = 12, 2 = 24, 3 = 72. D = 1210 m, S = 824 m sample. One sample, the 1^st^ timepoint of the 1210 m control was excluded due to low sequencing depth. Only OTUs greater than 0.005% of the total abundance were included in the plots.

The relative abundance of well-known oil degrading bacteria increased within the oil-amended microcosms. At the initial timepoint, *Proteobacteria* and *Verrucomicrobia*, were the most dominant phyla in the microbial communities from both depths (Figs 2a and S1a). *Gammaproteobacteria* of the phylum *Proteobacteria* has many well-known oil degrading species^4^ and was the one of the most abundant classes (Figs 2b and S1b). In the oil-amended microcosms from the 1210 m depth community, the bacterial population consisting mainly of *Gammaproteobacteria*, which made up almost 90% of the total community after 72 h. In the 824 m depth community, the *Proteobacteria* increased to 60% of the community in the oil-amended microcosms but increased to 95% in the oil with dispersant microcosms.

While the oil-degrading bacteria increased in relative abundance, the archaeal population decreased at both depths in the presence of oil. This decrease was observed in oil microcosms both with and without dispersant, especially after 72 h of incubation. Initially, the archaeal population constituted a large portion of the microbial community; *Euryarchaeota* and *Crenarchaeaota* accounted for more than 55% at 824 m and 40% at 1210 m. (Despite *Crenarchaeaota* being recently renamed *Thaumarchaeota*, we will use the classification and nomenclature of the Greengenes database version 13.5 in this paper where *Crenarchaeaota* is a phylum.) *Crenarchaeaota* alone accounted for more than 40% at the depth of 824 m and 25% at 1210 m. At the class level of taxonomy, *Thaumarchaeota* of the phylum *Crenarchaeaota* was the most abundant class and accounted for more than 15% at 1210 m and 40% at 824 m (Fig. 2b). After the final time points, the abundance of archaea consisting mainly of *Crenarchaeaota*, which comprised only about 10% of the control treatment. In the oil-amended treatments with and without dispersant after 72 h, the abundance of archaea decreased to below 2%, and *Proteobacteria* dominated the entire community. Archaea groups decreased at different rates during the incubation (Fig. S1c and d). The class *Thermoplasmata* of the phylum *Euryarchaeota* disappeared faster than the class *Thaumarchaeota* of the phylum *Crenarchaeota*.

The addition of dispersant increased the relative abundance of oil-degrading bacteria even more than oil alone, but decreased the relative abundance of archaea. The *Gammaproteobacteria* increased to higher levels in the oil plus dispersant conditions compared to the oil alone. The difference is very minor in the 1210 m but more dramatic in the 824 m samples. The *Gammaproteobacteria* increased by more than 20% in the oil with dispersant microcosms compared to the oil only microcosm.

**Figure 3:**
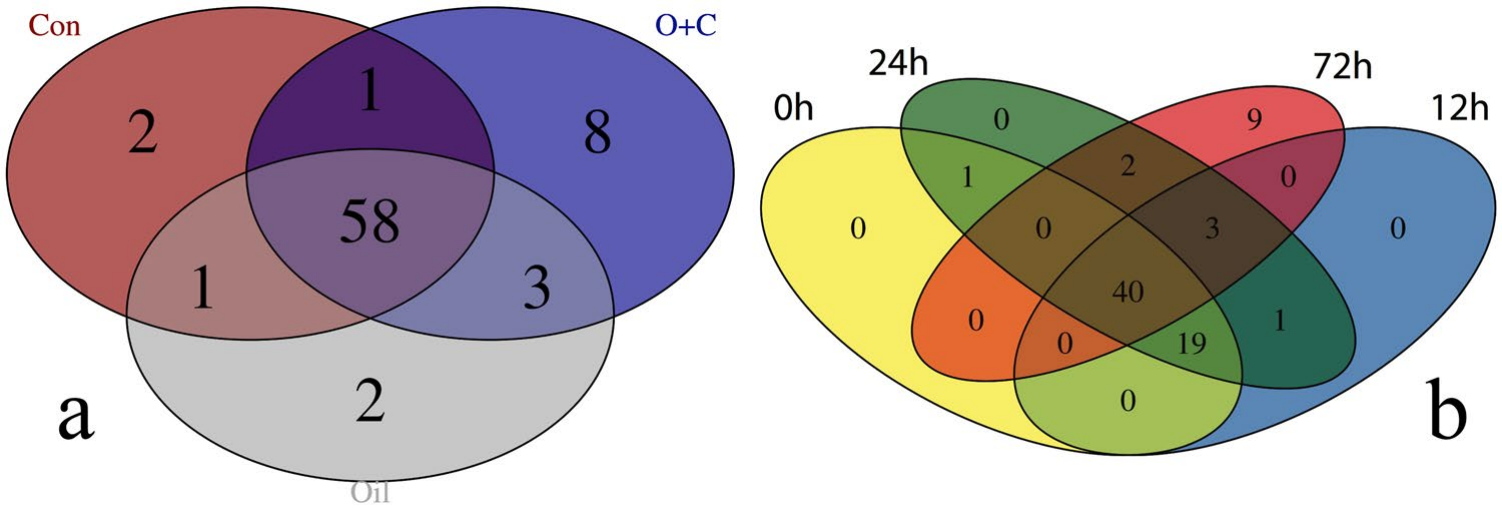
Venn diagrams of shared dominant OTUs between samples (**a**) Shared OTUs between microcosm treatments, (**b**) Shared OTUs between timepoints. The top 75 most abundant OTUs were included in the Venn diagram analysis. Values in the overlapping regions indicate the number of shared OTUs between sample types. C = control, O = oil-amendment, OC = oil with dispersant.

To determine the dominant OTUs across different treatments and time, we identified the overlap and distribution of the 75 most abundant OTUs in all samples (Fig. 3a and b). The majority of the OTUs, 58 of the total 75, were found in all treatments. The oil with dispersant microcosm had the highest number of distinct OTUs; 8 distinct OTUs were present in oil with dispersant. Likewise, 40 OTUs of the total 75, were present at all time points. For the most part, all the timepoints had common OTUs except for the last timepoint at 72 h, which had 9 distinct OTUs.

#### Microbial alpha diversity

**Figure 4:**
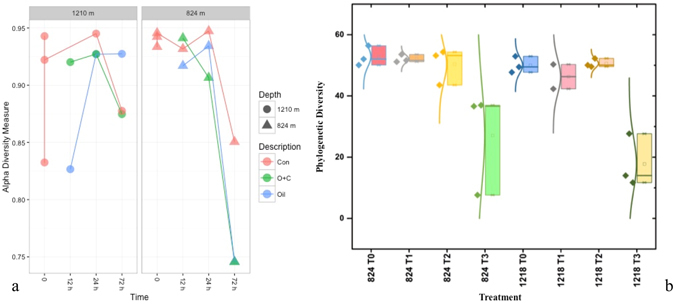
Alpha diversity. (**a**) Shannon diversity, (**b**) Phylogenetic diversity. The phylogenetic diversity results are grouped by the timepoint and depth. Points represent the control, oil and oil with dispersant microcosms for the given timepoint and depth. Boxplot and curved line next to it summarizes information about the range, sample distribution, and average phylogenetic diversity.

Alpha diversity metrics of species richness and evenness (Observed Species, Chao1, Shannon, and phylogenetic diversity) were determined for each treatment and timepoint (Figs 4a,b and S2a,b). As a general trend, the diversity declined dramatically over the course of the incubation. The decrease in diversity was seen in all treatments, even in the control microcosms. The control microcosms lost nearly half of their species richness after 72 h. The Chao1 and Shannon index, which considers both richness and evenness, also changed over time for the control microcosms. Of the three treatments, the application of oil and dispersant typically led to the lowest alpha diversity at both depths. On average, the diversity of the oil and dispersant community was less than half of its initial value.

#### Beta diversity

**Figure 5:**
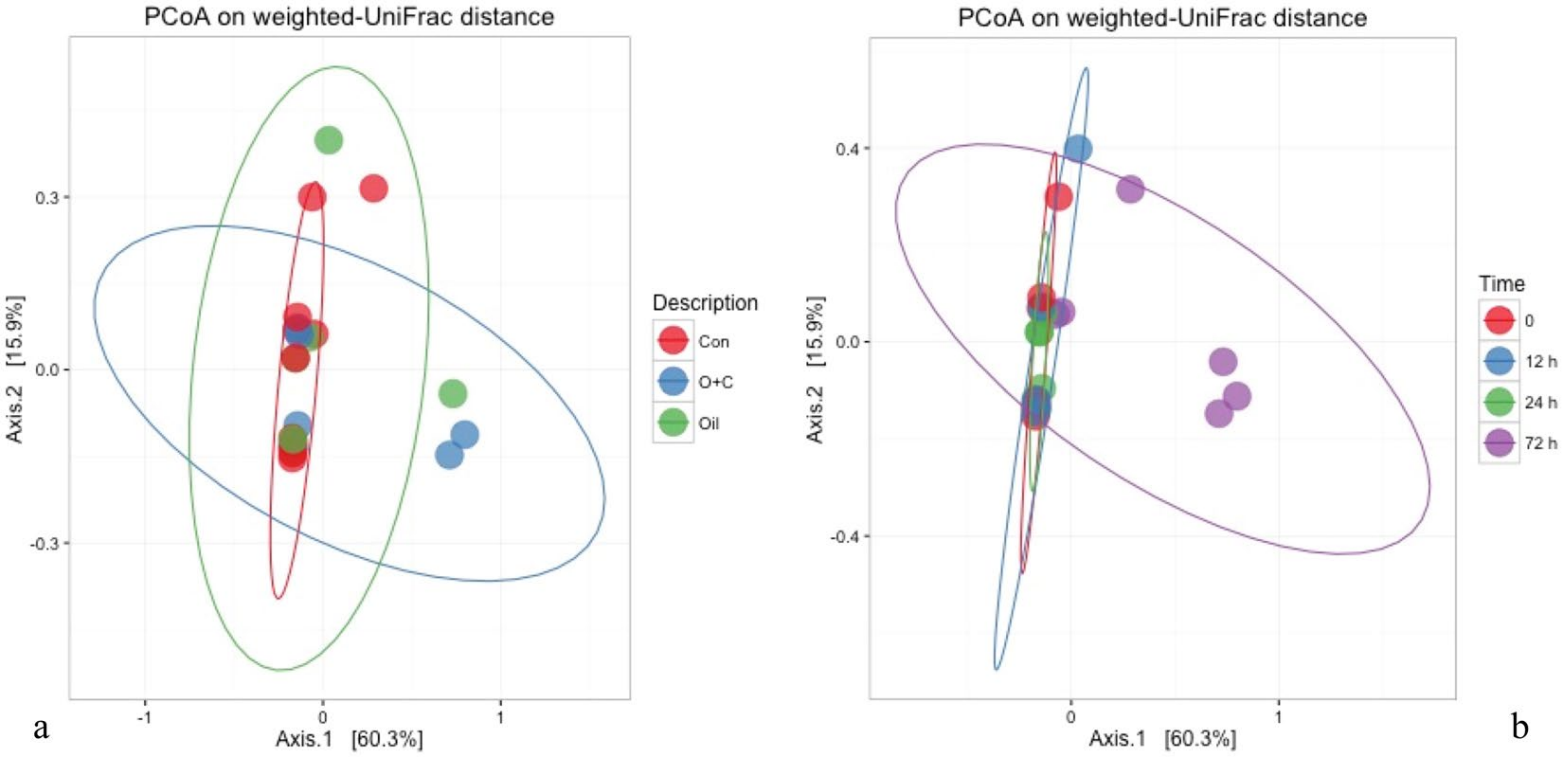
Beta Diversity. (**a**) Principal Coordinate Analysis (PCoA) of 16S rRNA gene sequencing results using weighted UniFrac distance grouped by treatment, the confidence ellipses represent the 95% confidence interval for each treatment. (**b**) PCoA results grouped by timepoint, the confidence ellipses represent the 95% confidence interval for each treatment.

Beta diversity analysis was used to determine the impact of treatments on the overall microbial community composition in all microcosms and the relationship among individual samples (Fig. 5). Principal Coordinate Analysis (PCoA) using a weighted UniFrac distance revealed that both incubation time and the amendment of oil and dispersant can greatly influence the microbial community (Figs 5a and S3a). Despite some variance in the time 0 samples at each depth and some outliers observed after 12 h, most communities in the early time phases (0, 12, and 24 h) were grouped very closely, indicating high similarity (Fig. 5b). After 72 h, more variance was found between control and other treatments, especially the treatment with oil and dispersant. The PCoA analysis performed on the bacterial and archaea communities also depicted a similar trend to what was observed in the whole microbial community (S3b and S3c).

#### Microbial Community Structure Changes in Response to Oil and Dispersant based on 16S rRNA gene amplicon sequencing

**Figure 6:**
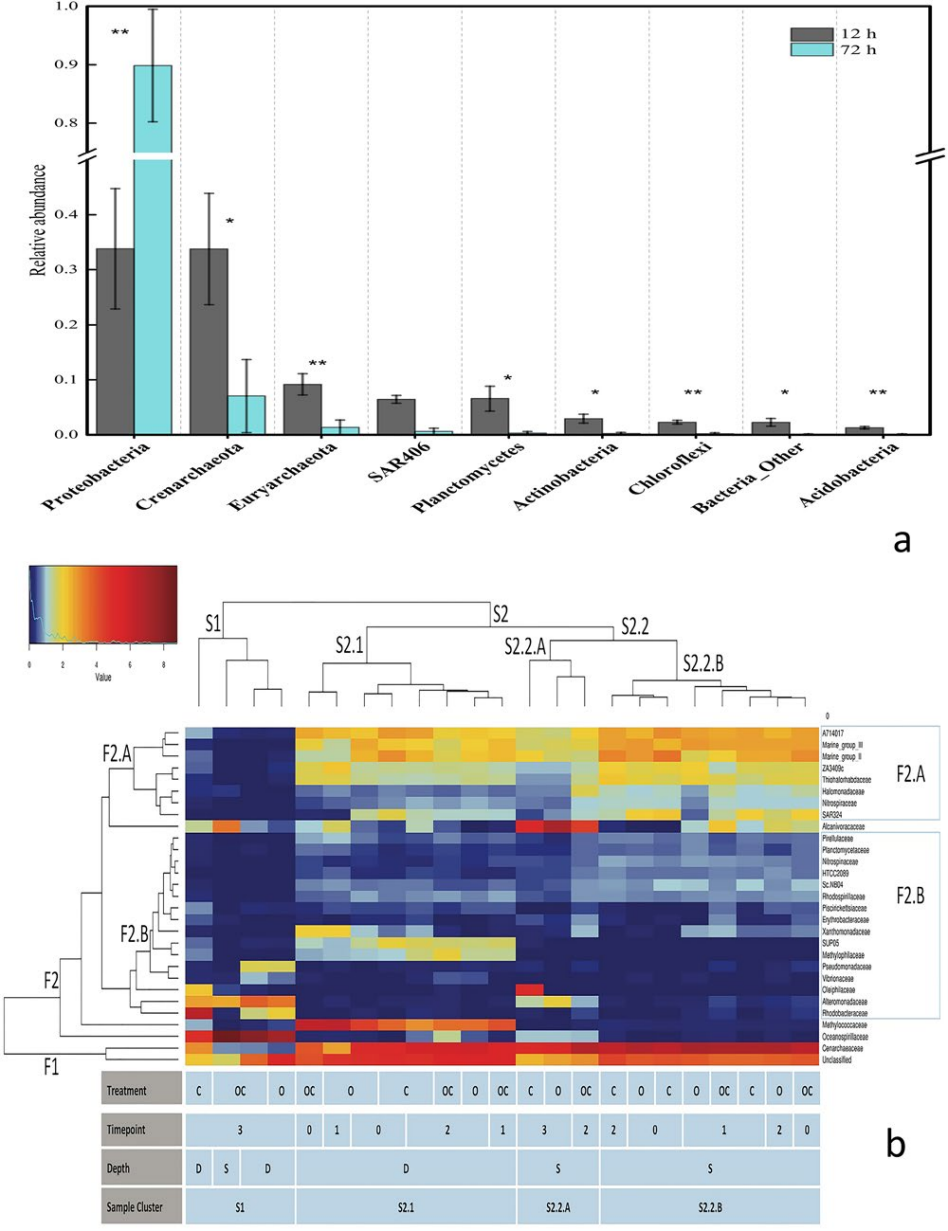
Abundance and distribution analysis of the dominant taxonomic groups. (**a**) The average abundance of the dominant Phylum in oil/oil with dispersant microcosms between 12 h and 72 h. Taxa differences were analyzed by two-tailed paired t-test (*P < 0.05 and FDR adjusted P < 0.1, **P < 0.05 and FDR adjusted P < 0.05). Error bars represent standard error (n = 4). (**b**) Heatmap and hierarchical clustering. Microcosms clustered based on taxa co-occurrence of the most abundant 29 taxonomic families.

The dominant taxonomic phylum and family (relative abundance >1%) in the oil-amended microcosms both with and without dispersant were statistically compared at 12 and 72 h using paired sample t-test (Figs 6a and S4 and S Tables 1 and 2). At the phylum level, *Proteobacteria* was greatly enriched during the incubation, which was prevalent in the GOM after DWH oil spill^16, 17, 19^. Among bacteria, the abundance of *Proteobacteria* (P = 0.00532, FDR = 0.0156) increased more than 2.5-fold from the first to last timepoint, while *Chloroflexi* (P = 0.00529, FDR = 0.0238) and *Acidobacteria* (P = 0.0065, FDR = 0.0146) showed a much lower abundance after that. Among archaea, the relative abundance of *Euryarchaeota* (P = 0.0274, FDR = 0.0492) dramatically decreased after 72 h of incubation. At the family level, we found that 1/3 of the most abundant families with a notable change (P < 0.05, FDR < 0.05) after 72 h.

To complement the beta diversity analysis and paired-sample t-test, hierarchical clustering of the samples’ microbial communities at the family taxonomic level provided more detailed information about notable population shifts (Fig. 6b). The samples clustered into two main groups based on the top 29 taxonomic families in the dataset. After 72 h, all the deeper 1210 m microcosms and the shallower 824 m oil with dispersant microcosms grouped into one cluster (S1). Other samples were grouped into two sub-clusters based on depth (S2.1 and S2.2a,b), indicating distinct abundance profiles of dominant taxa between most of the 824 m and 1210 m samples.

Treatments with oil did not cluster closely together. Even after 72 h, microcosms amended with both oil and dispersant did not cluster together with the microcosms amended with just oil. The large differences between the two treatments are due to the amendment of dispersant. After 72 h, the oil microcosm from the 824 m depth was more similar to its respective unamended control than the oil with dispersant microcosm.

The abundance of the *Cenarchaeaceae* family had a very distinct distribution pattern among microcosms compared to other families. It was high in the earlier microbial communities (S2), but very low in S1 cluster consisting of just 72 h samples. Similar to *Cenarchaeaceae*, families included in the cluster F2.a also had higher abundance in the sample group of S2.1 and S2.2 than S1. The F2.a cluster abundance was lower in S2.2.a than in S2.1 and S2.2.b.

Both *Oceanospirillaceae a*nd *Alteromonadaceae* had a larger population in the later timepoint samples of cluster S1 than the earlier timepoints of S2. These families contain known oil-degrading species, which were found in high abundance in the GOM after the DWH oil spill^16, 17, 23^. Within the cluster of S1, treatments with dispersant, there was a higher relative abundance of *Oceanospirillaceae*. These two taxonomic families also had a modest presence in the 72 h control and oil only microcosm of S2.2a. The oil with dispersant sample incubated for only 24 h did not have a high abundance of *Oceanospirillaceae a*nd *Alteromonadaceae*, indicating the importance of time for these taxa to respond.

#### Microbial community functional structure changes based on GeoChip metagenomics Microbial functional composition

**Figure 7:**
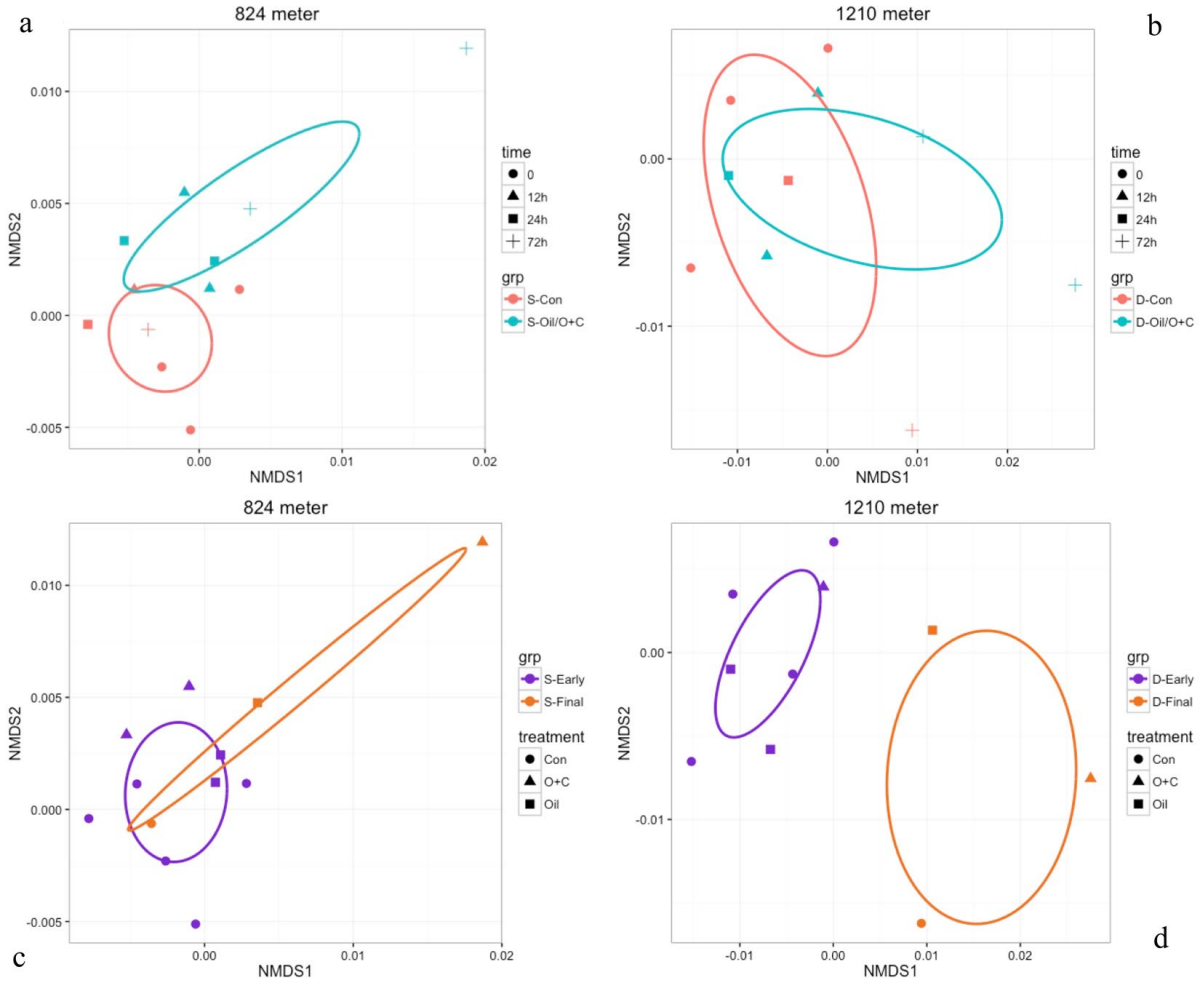
Non-metric Dimensional Scaling (NMDS) plot of *GeoChip* 5.0 functional genes related to organic bioremediation a) 824 m samples with vs without oil. (**b**) 1210 m samples with vs without oil. (**c**) 824 m samples at the first 3 timepoints vs final timepoint. (**d**) 1210 m samples at the first 3 timepoints vs final timepoint. In Fig. 7a and b, red = control, turquoise = all oil-amended microcosms (both “oil” and “oil with dispersant” microcosms). In Fig. 7c and d, purple = earlier timepoints (0, 12 h and 24 h). Brown represents samples from the final timepoint at 72 h. In all plots, the shape of the point indicates the timepoint: circle for initial, triangle for 12 h, square for 24 h, and cross for 72 h. The ellipses are 95% confidence intervals.

The *GeoChip* 5 detected a substantial number of genes related to organic compound bioremediation such as genes encoding for degradative enzymes for polycyclic aromatics and other hydrocarbons, BTEX and other diverse organic compounds. Non-metric multidimensional scaling (NMDS) analysis with Bray-Curtis dissimilarity was used to assess the differences in bioremediation related genes between samples (Fig. 7a). The stress value for the NMDS was 0.09043914, suggesting the ordination is reasonably accurate. Two samples were omitted from the ordination due to extraordinarily high dissimilarity, the 12 h control and 24 h oil with dispersant microcosms at 1210 m.

The addition of oil led to large differences in the microbial community functional structure, particularly in the 824 m samples. The bioremediation genes of the controls and oil-amended microcosms (both with and without dispersant) were significantly different based on an adonis test using the vegan package in R (R^2^ = 0.17807, P < 0.05). The addition of oil caused a larger difference in bioremediation gene abundance within the shallower 824 m samples (Fig. 7a and b).

The majority of the differences in bioremediation genes were only observed after 72 h (Fig. 7c and d). The 95% confidence ellipses show that the variance between samples of earlier timepoints were much less than the last timepoint. In other words, the samples from 0, 12 and 24 h have relatively few differences. After incubation, all 72 h 1210 m microcosms showed a very clear difference compared to earlier 1210 m microcosms. In contrast, we observed less difference among 824 m microcosms except for the oil and dispersant treatment.

#### Core carbon degradation and organic bioremediation gene analysis

**Figure 8:**
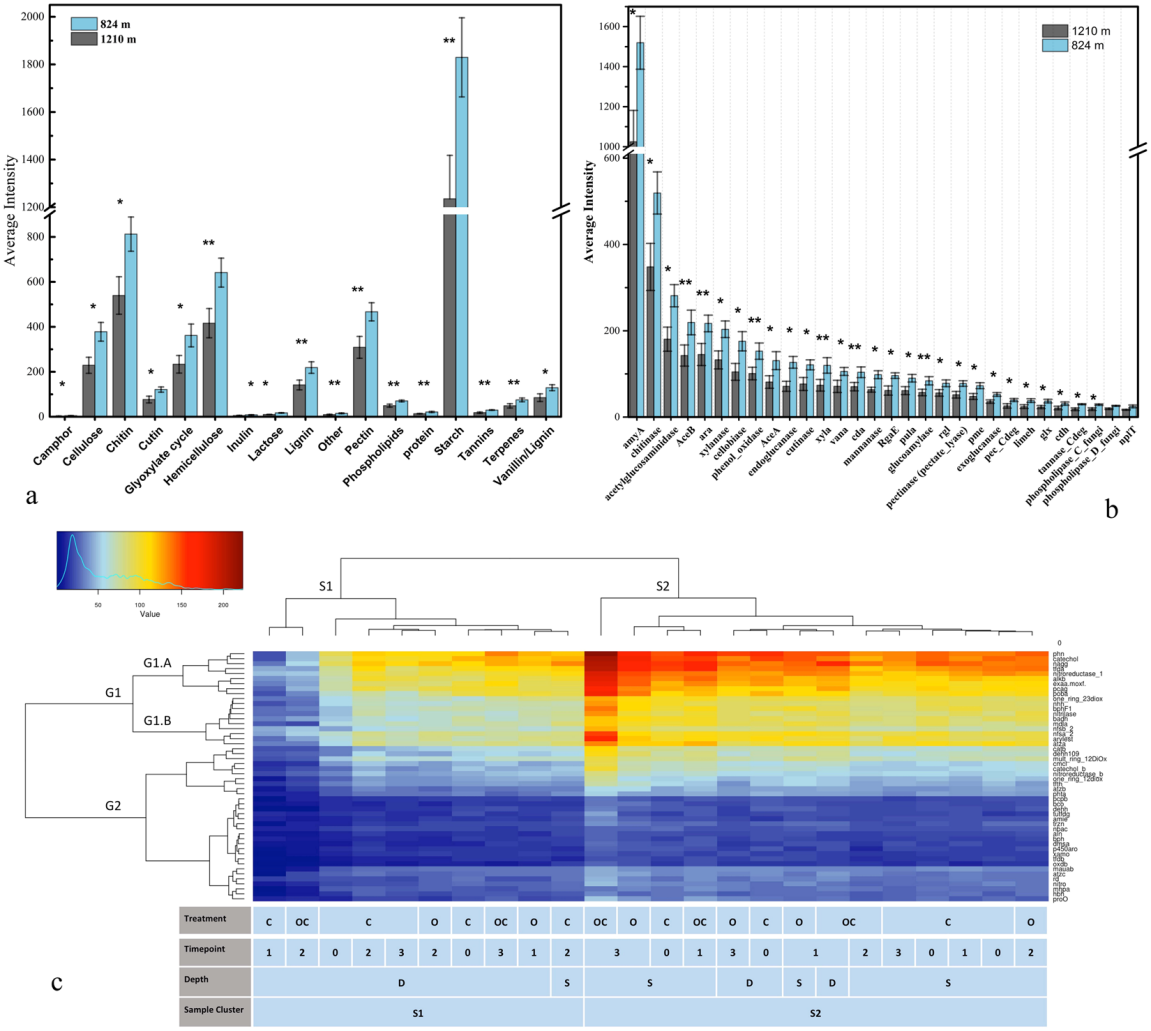
The intensity and distribution analysis of functional genes. The average of (**a**) overall carbon degradation groups and (**b**) the most abundant 30 carbon degradation genes. The differences of gene intensity in oil/oil with dispersant microcosms between 824 m and 1210 m were analyzed by one-tailed independent t-test (*P < 0.05 and FDR adjusted P < 0.1, **P < 0.05 and FDR adjusted P < 0.05). Error bars represent standard error (n = 4). (**c**) Heatmap and hierarchical clustering of the 50 most abundant aromatic bioremediation genes.

Carbon compounds are a major constituent of crude oil. Therefore, the abundance of genes related to carbon compound degradation and organic remediation is a key index for oil biodegradation. To clarify the details of the functional potential differentiations in different depths of water column, we focused on the genes related to carbon degradation and organic remediation (Fig. 8a,b and S Tables 3, 4 and 5). Based on previous studies of relatively lower oil biodegradation in deep ocean DWH oil spill^2–4, 16–19^ and strong stratification of microbial community in E. Med. by depth and nutrient^6, 24^, we hypothesized that microbial community in shallower E. Med. water column had a stronger response to crude oil contamination than the deeper E. Med. water column. Independent-sample t-test was applied to determine oil’s influence on these genes between the 824 m and 1210 m oil amended microcosms combining both treatments with and without dispersant at these two time points.

The gene intensity of each category under carbon degradation was higher in 824 m than 1210 m. Among them, we found much higher enrichment of genes related to starch (P = 0.0263, FDR = 0.0466), hemicellulose (P = 0.0247, FDR = 0.0493), lignin (P = 0.0308, FDR = 0.0491), pectin (P = 0.0233, FDR = 0.044), phospholipids (P = 0.0187, FDR = 0.0414), protein (P = 0.0168, FDR = 0.0402), tannins (P = 0.00683, FDR = 0.0488), and terpenes (P = 0.0383, FDR = 0.0488) degradation in 824 m than 1210 m microcosms. In the analysis of the most abundant 30 carbon degradation genes, the *GeoChip* results showed five genes (P < 0.05, FDR < 0.05) with a dramatically higher intensity in 824 m compared to 1210 m microbial community. The analysis of the most abundant 70 genes related to organic remediation also showed higher average intensity in 824 m than 1210 m samples, but the difference between depth was not as much as carbon degradation genes.

To identify the most differentially abundant functional genes, the 50 most abundant bioremediation genes were clustered using hierarchical clustering (Fig. 8c). All samples were clustered into two major groups (S1 and S2), while the 50 genes were clustered into two groups (G1 and G2) with 2 notable sub-groups (G1.a, G1.b). Two samples, the 1210 m 12 h control and 24 h “oil + dispersant” microcosms, had extremely low total abundances, which strongly suggests that they were outliers and would be difficult to compare them to other samples.

Samples in S2 usually had a higher abundance of gene cluster G1.a and G1.b. Of all the samples, the 72 h 824 m community with oil and dispersant microcosm had the highest relative abundance of multiple bioremediation genes in G1.a and G1.b. G1.a includes many aromatic and hydrocarbon degradation genes like *alkb*, *nitroreductase_1*, *pcag*, *poba*, *nag*, *catechol*, *tlda*, and *arylest*.

## Discussion

The goal of this study was to characterize the response of a deep ocean microbial communities to crude oil and chemical dispersants using 16S rRNA amplicon sequencing and *GeoChip* metagenomics. In this study, the microbial communities from the E. Med. Deep water at 824 m and 1210 m exhibited a similar response to oil contamination. Both seawater samples became enriched with oil-degrading species after 72 h. The amendment of dispersant increased the relative abundance of oil-degrading species at the 824 m microcosm after 72 h, more analysis and replication are needed to test the potential selection of dispersant for oil degrading bacteria in deep ocean^16, 17^. The rapid bloom of oil-degrading species from the indigenous microbial community has important implications for a robust oil biodegradation potential. These microbes may aid oil bioremediation in the E. Med. if a marine oil spill should ever occur.

The most enriched bacterial taxa within oil microcosms were from *Oceanospirillaceae* family in the phylum of *Proteobacteria*. Since *Oceanospirillaceae* were also dominant species after the DWH oil spill^15, 16^, it seems to be a highly ubiquitous oil-degrading species capable of degrading oil under very different environmental conditions. This is one of the first studies of similar oil degrading bacterial groups proliferating in a deep-water basin other than GOM immediately after oil contamination.

The archaeal population decreased in all the treatments, especially in the presence of oil and dispersant after 72 h. A similar decrease in archaea was also observed in the GOM after the DWH oil spill in both field samples collected from the open ocean and within laboratory microcosm experiments^16, 19^. The drastic accelerated decrease in archaeal populations at both sites strongly suggest that the bacteria have a competitive advantage over archaea for crude oil and dispersant, despite some archaea also having the capacity to utilize compounds from oil^3^.

In addition to changing the phylogenetic composition of the microbial community, oil amendment can also increase the abundance of many important genes involved in carbon degradation and organic remediation. After the DWH oil spill in the GOM, many genes related to aromatic and aliphatic biodegradation were also enriched in the microbial communities from both surface and deep water, meanwhile strong biodegradation was detected of those compounds^16, 17, 25^.

The application of dispersant can strongly stimulate oil degrading bacteria and deplete archaea at both depths after 3 days, especially in the 824 m microbial communities. The abundance of *Proteobacteria* was much higher when dispersant was added into the 824 m samples. The addition of dispersant can greatly decrease the community diversity leading to a simplified community. Although there are some conflicting reports about the effect of dispersant on microbial communities^22^, our results show that dispersant addition could increase the relative abundance of oil degraders in E. Med. In addition, many important genes related to organic remediation were enriched in the 824 m 72 h microcosm when dispersant was added, suggesting a greater oil degradation potential. Our results are consistent with studies carried out in the GOM where dispersant can increase the oil biodegrading population and its applicability in E. Med.^18, 19, 26^.

Despite only slight differences in the geochemical features, the microbial communities at two depths showed differential response to crude oil and the application of dispersant, especially in microbial functional structure. The 824 m and 1210 m microbial communities had similar phylogenetic composition of well-known oil degraders such as *Oceanospirillaceae*, *Alteromonadaceae* and *Alcanivoraceae* after 72 h. However, the 824 m communities had a much higher abundance of genes for carbon degradation and organic bioremediation than 1210 m, indicating a better oil biodegradation potential. This is one of the first times different functional gene composition have been found between different depths of deep seawater column. Previous studies have only compared oil degradation capacity at a much broader scale of resolution between surface and deep water^18^. Because of the differences between two depths that are only 400 m apart, future work in deep water oil degradation should study various depths of deep marine water rather than just one depth.

Another finding from our study was the storage effects on the microbial community structure over time. In our control group, we observed drastic community changes after 3 days of incubation. Similar storage bottle effects have been reported^27^, but there is very limited information on exactly how storage effects can affect 16S rRNA community composition and functional gene structure, and how fast these change can occur. Our study suggests that storage times longer than 72 h can tremendously change microbial phylogenetic and functional community structure, causing the microcosms to be unrepresentative of the natural community, and therefore lead to bias and inaccurate results^22^.

In the future, more quantitative analysis should be used to characterize the microbial activity such as the carbon utilization rate, the cell abundance before and after introduction of oil and the microbial respiration over a longer incubation time. Some of these analyses were unfeasible on board the ship for this study. It would also be beneficial in future experiments to more thoroughly investigate the influence of dispersant alone on the microbial community and the microbial response in water samples collected in different seasons. Also, because of the low nutrient levels in E. Med.^6, 24^, it would be worthwhile to determine if extra nutrients in addition to oil would stimulate more oil degrading bacteria. This could be very practical and meaningful in the event of an oil spill when biostimulation became necessary^4^. Multiple lines of evidence will be helpful for more accurate results.

In conclusion, our study demonstrated that oil contamination can dramatically increase the abundance of hydrocarbon degrading bacteria in the deep Mediterranean, which shows their strong oil degradation potential. The rapid adaption of the microbial community to the introduction of oil was similar to the deep community in the GOM. Like the GOM, oil amendment caused the enrichment of the 16S rRNA gene amplicon sequences of various known oil degrading bacteria, especially *Oceanospirillaceae*, and multiple functional genes related to aromatic and aliphatic hydrocarbon biodegradation occurred in the Mediterranean Sea. The amendment of dispersant led to an enhanced enrichment of these oil degrading microorganisms in the community, indicating its applicability for oil spills. In addition, the deep microbial communities from shallower depth had more oil degrading genes enriched in the response to oil contamination and dispersant addition. Together these results can help us better our understanding of the microbial community adaption to oil contamination and provide research for more accurate estimation of the oil degradation potential in deep Mediterranean Sea.

## Methods

### Site Description and Sampling

Deep ocean water samples were collected in October 2012 from the E. Med. Sea. The sampling was carried out during BP’s West Nile Delta oceanographic survey aboard the MV Fugro Navigator. Water was collected from two depths at one station (Latitude: 31.8058° N and Longitude: 29.5683° E) using Niskin bottles. Water was collected from 824 m and 1210 m depths. Multiple environmental variables such as pH, temperature, and salinity were analyzed *in situ* with the Valeport Midas+ CTD. Geochemical parameters with microbial community analysis is described in detail by Techtmann *et al*.^6^.

Analog crude oil (Norne Blend) was provided by BP. The API gravity of the crude oil was 29.6°. The dispersant used in this study, COREXIT 9500 (Nalco, Sugar Land, TX), is the identical dispersant used during the DWH spill in 2010.

### Experimental Set-up

Immediately after sampling, on-ship microcosms were set up aerobically with 2 L seawater each. The incubation was carried out at 15 °C in the dark. Three different treatments were set up: ‘control’ (seawater), ‘oil’ (seawater and 10 ppm of oil), and ‘oil + COREXIT’ (seawater, 10 ppm of oil, and 0.167 ppm of COREXIT 9500). Destructive sampling was performed at 0, 12, 24, and 72 h. The sample was filtered through a 0.22 µm filter and stored at −20 °C until shipped to the lab for extraction. Because of the space limitation on board, only one enrichment was set up for each enrichment at different time phases.

### Genomic DNA extraction and PCR amplification

The genomic DNA was extracted using the modified Miller’s method^15^ and then cleaned using the Genomic DNA clean and Concentrator Kit (Zymo Research, Irvine CA). DNA quality was analyzed by NanoDrop (Thermo Scientific Waltham, MA) using ratios of 260/280 and 260/230. DNA concentration was determined by Picogreen (Life Technologies, Carlsbad CA). Then, universal primers 515f and barcoded 806r and Phusion DNA polymerase (Thermo Scientific Waltham, MA) were used for the PCR reaction to target the V4 region of the 16S rRNA gene. An additional 12-bp barcode index was included in the reverse primer to multiplex different samples for sequencing analysis^23^.

### 16S rRNA gene amplicon sequencing and data analysis

16S rRNA gene libraries and sequencing was performed on the Illumina MiSeq following the method described in Caporaso *et al*.^23^. To briefly summarize, the 16S PCR products were pooled together. The quality and sequence size was determined by Bioanalyzer (Agilent, Santa Clara CA). Another purification process was occasionally needed before dilution. Quantitative-PCR was used to obtain the final accurate concentration of the pooled amplicon libraries. The finalized libraries were sequenced by Illumina MiSeq platform with a V2 kit (Illumina, San Diego CA).

The resulting sequences were analyzed by the QIIME 1.8 pipeline^28^. The paired-end raw DNA sequences were assembled with the fastq-join script, demultiplexed and removed of any chimeras. Reads with phred score less than 20 were removed. The open reference clustering method was used to cluster the sequences into operational taxonomic units of 97% similarity. Sequences were aligned by PyNAST and assigned with Greengenes taxonomy using the database released in May 2013^29, 30^. Any operational taxonomic unit^31^ with less than 0.005% of the total abundance was removed to avoid the influence of spurious OTUs in later analysis. The statistical analyses of alpha diversity, beta diversity and differentially abundant taxa were carried out using Phyloseq in R^32^ and Calypso^33^ (hierarchical clustering and T-test). The two tailed paired-sample t-test was used to analyze the resulted sequencing data with an alpha value at 0.05 for both P-value and the FDR adjusted P-value. After that, the false discovery rate (FDR) controlling procedure based on Bonferroni correction was applied to adjust the type one error.

### GeoChip hybridization and data preprocessing

DNA for *GeoChip* analysis was also extracted using the modified Miller method^15^. The final DNA concentration was determined by Nanodrop (Thermo Scientific, Waltham, MA) and Picogreen (Life Technologies, Carlsbad CA). After multiple displacement amplification (MDA) and purification, the resultant product was labeled with Cy-5 fluorescent dye (GE Healthcare) with random primers. Before another purification, the labeled DNA was dried out at 45 °C for 45 min, followed by suspension in 120 µl hybridization buffer and denaturation at 95 °C for 5 min. The resulting DNA was then hybridized with a *GeoChip* 5.0 microarray at 42 °C for 12 h on an HS4800 Pro hybridization station (Tecan US, Durham, NC). The microarray was then washed with multiple washing buffers and scanned using a San Array Express Microarray Scanner. The signal intensity of each probe was detected by ImaGene 6.0 software (Biodiscovery Inc., EL Segundo, CA).

The resulting intensity was normalized and analyzed as previously described. Results were downloaded from the website (http://ieg.ou.edu/microarray/) and processed by the following steps: (i) probes with low detecting quality shown as 1 or 3 and with a signal to noise ratio (SNR) lower than 2 were discarded; all of the low-quality spots were removed based on the SNR ratio, so that the total percentage of negative control (thermophile) probes in each microarray was no more than 5%, (ii) after that, the signal intensity of each gene was transformed into natural logarithmic form and divided by the mean intensity of all genes on the microarray^17, 34^. The statistical analysis was also performed with the vegan package in R^35^ (Non-metric Dimensional Scaling analysis) and Calypso^33^ (hierarchical clustering and t-test analysis). The the one tailed independent-sample t-test was used to analyze the normalized *GeoChip* data with an alpha value at 0.05 for both P-value and the FDR adjusted P-value. After that, the FDR controlling procedure based on Bonferroni correction was applied to adjust the type one error.

## Electronic supplementary material

Supplementary Material
