# Supplementary material for: Rapid Response of Eastern Mediterranean Deep Sea Microbial Communities to Oil
Source: Sci Rep. 2017 Jul 18;7:5762. doi: 10.1038/s41598-017-05958-x (PMC5515914; doi:10.1038/s41598-017-05958-x)
Supplement: Supplementary file 1 — Supplementary Material [file 41598_2017_5958_MOESM1_ESM.pdf]

Supplementary Information

Rapid Response of Eastern Mediterranean Deep Sea Microbial Communities to Oil

Jiang Liu[1], Stephen M. Techtmann[2], Hannah L. Woo[3], Daliang Ning[5], Julian L. Fortney[3], Terry C. Hazen[1,3,4,6]

[1] Department of Microbiology, University of Tennessee Knoxville, Knoxville, TN

[2] Department of Biological Sciences, Michigan Technological University, Houghton, MI

[3] Department of Civil & Environmental Engineering, University of Tennessee, Knoxville, TN

[4] Department of Earth & Planetary Sciences, University of Tennessee, Knoxville, TN

[5] Consolidated Core Laboratory, Institute for Environmental Genomics and Department of Microbiology and Plant Biology, The University of Oklahoma, Norman, OK

[6] Biosciences Division, Oak Ridge National Lab, Oak Ridge, TN

Corresponding author:
Terry C. Hazen
The University of Tennessee
676 Dabney Hall
Knoxville, Tennessee 37996-1605
Phone: 865-974-7709
E-mail: tchazen@utk.edu

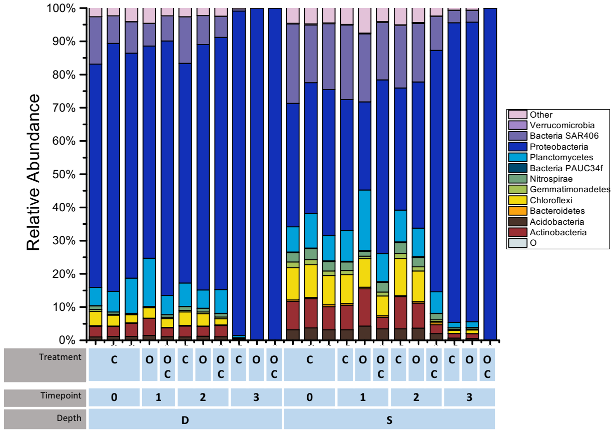
Relative Abundance
Other
Verrucomicrobia
Bacteria SAR406
Proteobacteria
Planctomycetes
Bacteria PAUC34f
Nitrospirae
Gemmatimonadetes
Chloroflexi
Bacteroidetes
Acidobacteria
Actinobacteria
O
Treatment
Timepoint
Depth
D
S

a

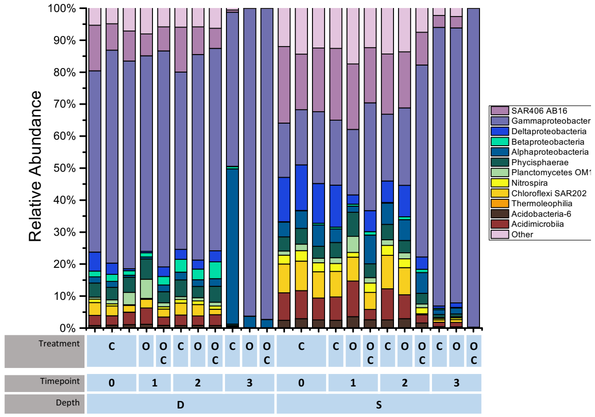
Relative Abundance
SAR406 AB16
Gammaproteobacter
Deltaproteobacteria
Betaproteobacteria
Alphaproteobacteria
Phycisphaerae
Planctomycetes OM1
Nitrospira
Chloroflexi SAR202
Thermoleophilia
Acidobacteria-6
Acidimicrobiia
Other
Treatment
Timepoint
Depth
D
S

b

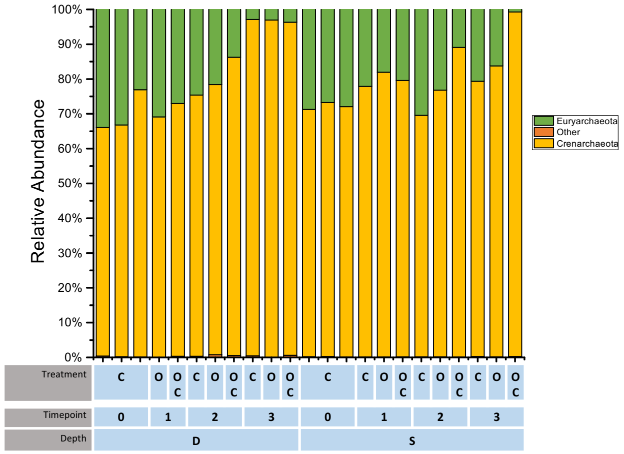
Relative Abundance
Euryarchaeota
Other
Crenarchaeota
Treatment
Timepoint
Depth
D
S

c

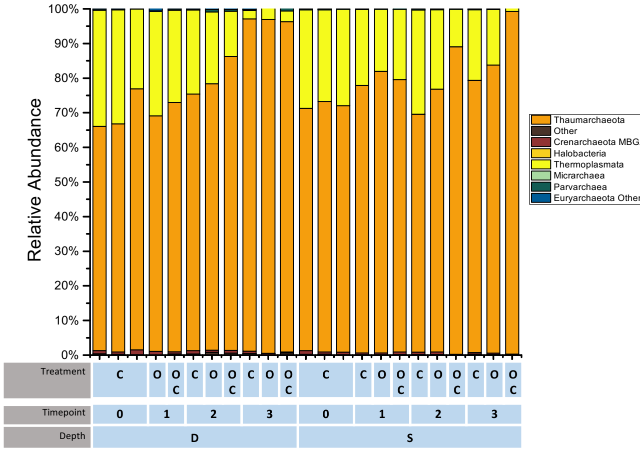
Relative Abundance
Thaumarchaeota
Other
Crenarchaeota MBG
Halobacteria
Thermoplasmata
Micrarchaea
Parvarchaea
Euryarchaeota Other
Treatment
Timepoint
Depth
D
S

d

Figure S1. Taxa Plots of the Microbial community. a) Bacterial Microbial Community at the Phylum Level, b) Bacterial Microbial Community at the Class level, c) Archaeal Microbial Community at the Phylum, d) Archaeal Microbial Community at the Class level. Plots show the relative abundance of the 16S rRNA sequencing gene amplicon sequencing OTUs summarized at the phylum and class level for the bacteria and archaea separately. The samples are labeled with their treatment, timepoint and depth. The treatments are labeled C for control, O for oil-amendment, OC for oil with dispersant. The timepoints are 0 for initial, 1 for 12 h, 2 for 24 h and 3 for 72 h. The two depths are labeled D for the deeper 1210 m sample and S for the shallower 824 m sample. One sample, the 1st timepoint of the 1210 m control was excluded due to low sequencing depth. Only OTUs greater than 0.005% of the total abundance were included in the plots.

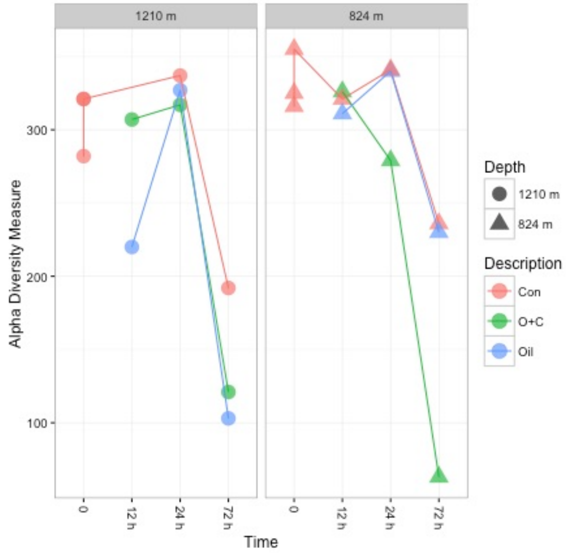

a

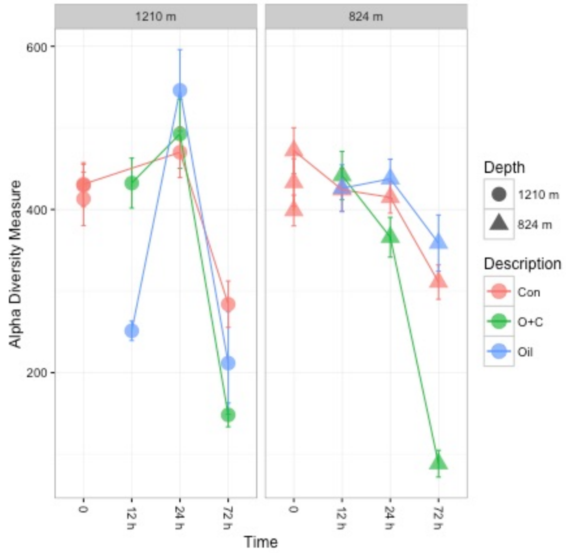

b

Figure S2 Alpha diversity. a) Observed number of species, b) Chao1 diversity, Alpha diversity metrics were calculated from the 16S rRNA gene amplicon sequencing results using the R package, vegan.

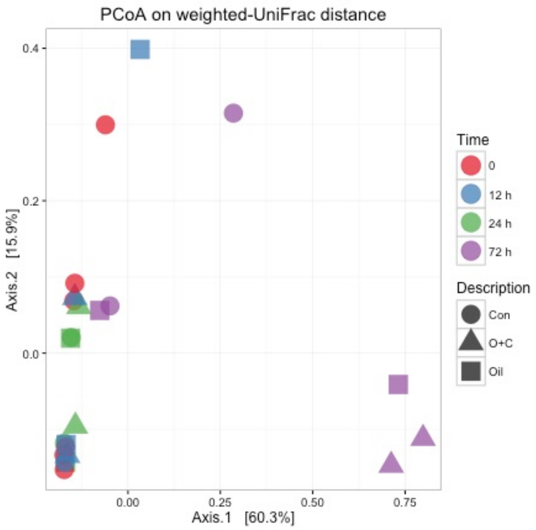
PCoA on weighted-UniFrac distance
Axis.2 [15.9%]
Axis.1 [60.3%]
0.4
0.2
0.0
0.00
0.25
0.50
0.75
Time
0
12 h
24 h
72 h
Description
Con
O+C
Oil

a

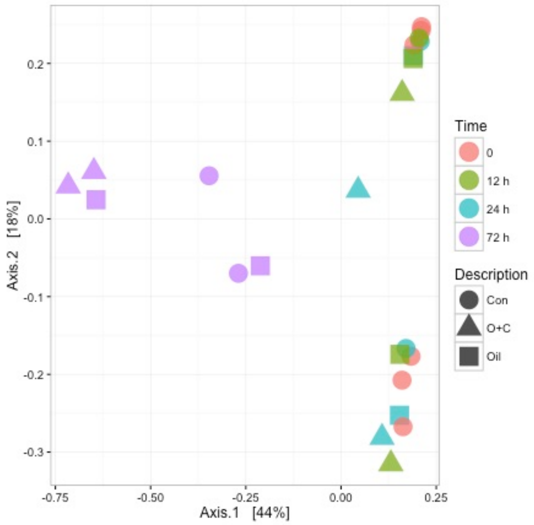
Axis.2 [18%]
Axis.1 [44%]
0.2
0.1
0.0
-0.1
-0.2
-0.3
-0.75
-0.50
-0.25
0.00
0.25
Time
0
12 h
24 h
72 h
Description
Con
O+C
Oil

b

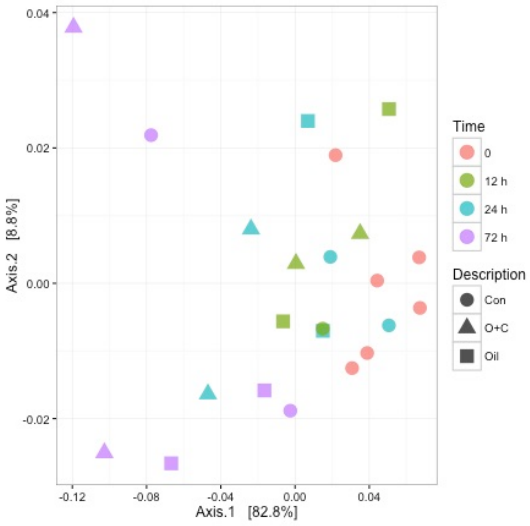
Axis.2 [8.8%]
Axis.1 [82.8%]
0.04
0.02
0.00
-0.02
-0.12
-0.08
-0.04
0.00
0.04
Time
0
12 h
24 h
72 h
Description
Con
O+C
Oil

c

Figure S3. Beta Diversity. a) Principal Coordinate Analysis (PCoA) of 16S rRNA gene sequencing results using weighted UniFrac distance, **b) Bacterial PCoA using weighted UniFrac**, Only bacterial OTUs were included in this PCoA analysis. c) **Archaeal PCoA using weighted UniFrac**. Only archaeal OTUs were included in this PCoA analysis.

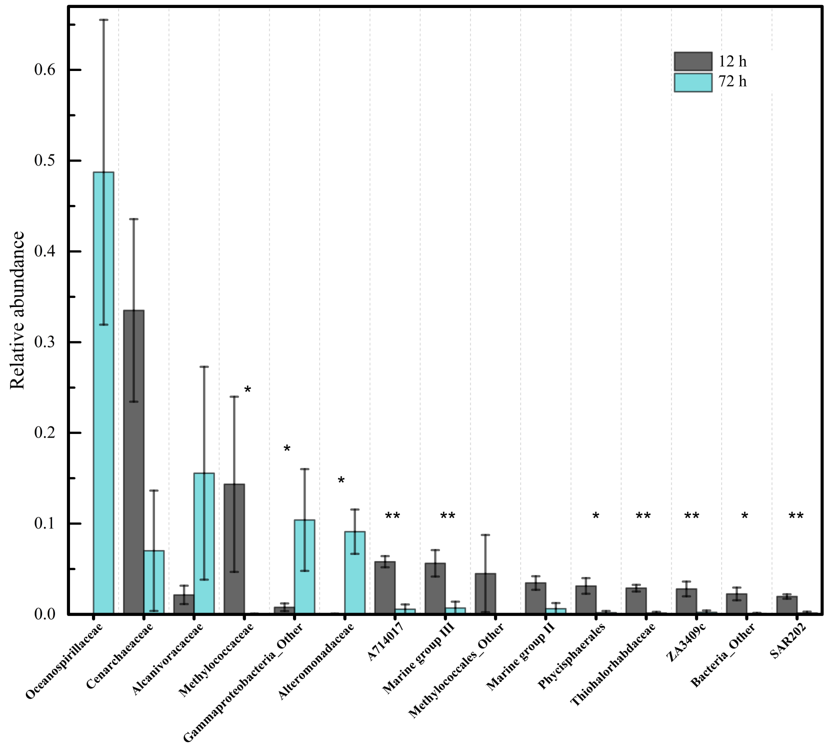

Figure S4. The average abundance of the dominant Phylum in oil/oil with dispersant microcosms between 12 hour and 72 hour. Taxa differences were analyzed by two-tailed paired t-test (*: P< 0.05 and FDR adjusted P< 0.1, **: P< 0.05 and FDR adjusted P< 0.05). Error bars represent standard error (n= 4).

Phylum	P value	Ave 12 h	Standard Error 12 h	Ave 72 h	Standard Error 72 h	Degree of freedom	Alpha	t-statistic	FDR adjusted P value
**Proteobacteria**	0.005321605	0.33835	0.109569746	0.89855	0.096184454	3	0.05	-7.293401186	0.015964815
**Crenarchaeota**	0.047874712	0.33795	0.101329014	0.0703	0.06643626	3	0.05	3.23934576	0.061553201
**Euryarchaeota**	0.027359332	0.0917	0.019510083	0.0134	0.01326675	3	0.05	4.035957367	0.049246798
**SAR406**	0.001344004	0.06415	0.007065114	0.0062	0.005800575	3	0.05	11.69276325	0.012096034
**Planctomycetes**	0.071397651	0.0656	0.022720182	0.00315	0.00288603	3	0.05	2.739010938	0.071397651
**Actinobacteria**	0.033429737	0.0292	0.008061431	0.00235	0.002091849	3	0.05	3.736290717	0.050144605
**Chloroflexi**	0.005289049	0.0228	0.003381321	0.0019	0.001833939	3	0.05	7.309002987	0.02380072
**Bacteria Other**	0.048585294	0.0226	0.006969457	0.0009	0.000834666	3	0.05	3.219972078	0.054658456
**Acidobacteria**	0.006500155	0.0128	0.002118175	0.00095	0.00088459	3	0.05	6.801103558	0.01462535

Table S 1. **The detailed results of two-tailed paired t-test on dominant phylum in oil/oil with dispersant microcosms between 12 hour and 72 hour (t-test details of Fig. 6a).** The FDR controlling procedure based on Bonferroni correction was applied to adjust the type one error.

Family	P value	Ave 12h	Standard Error 12 h	Ave 72h	Standard Error 72 h	Degree of freedom	Alpha	t-statistic	FDR adjusted P value
**Oceanospirillaceae**	0.062600003	0.0003	5.7735E-05	0.48725	0.168056605	3	0.05	-2.898078151	0.93900004
**Cenarchaeaceae**	0.047800141	0.33495	0.100637911	0.0701	0.066236269	3	0.05	3.241399977	0.358501058
**Alcanivoracaceae**	0.342175	0.02145	0.010114799	0.1556	0.117234381	3	0.05	-1.125818778	1.710874998
**Methylococcaceae**	0.233961939	0.14335	0.096501861	0.0005	0.000378594	3	0.05	1.486038634	0.877357271
**Gammaproteobacteria Other**	0.182660644	0.00785	0.004321555	0.10405	0.056036142	3	0.05	-1.726785801	0.547981933
**Alteromonadaceae**	0.034656555	0.0006	0.00033665	0.0911	0.024471684	3	0.05	-3.684172262	0.086641387
**A714017**	0.001710859	0.05805	0.006206113	0.0057	0.005300629	3	0.05	10.77246393	0.003666127
**Marine group III**	0.025823728	0.05625	0.014553665	0.007	0.007	3	0.05	4.125578305	0.04841949
**Methylococcales Other**	0.369161756	0.04495	0.042578506	0.00015	9.57427E-05	3	0.05	1.054278172	0.615269593
**Marine group II**	0.078177097	0.0346	0.007515761	0.00625	0.006183513	3	0.05	2.632203055	0.117265645
**Phycisphaerales**	0.04402839	0.0313	0.008593602	0.00195	0.001883923	3	0.05	3.350931902	0.060038714
**Thiohalorhabdaceae**	0.002663764	0.02895	0.003680919	0.00145	0.001384136	3	0.05	9.261481915	0.003329705
**ZA3409c**	0.039000337	0.028	0.008178834	0.0023	0.002042058	3	0.05	3.517029815	0.045000388
**Bacteria Other**	0.048585294	0.0226	0.006969457	0.0009	0.000834666	3	0.05	3.219972078	0.052055673
**SAR202**	0.003769155	0.0197	0.002444722	0.00155	0.001484082	3	0.05	8.219628766	0.003769155

Table S 2. **The detailed results of two-tailed paired t-test on dominant families in oil/oil with dispersant microcosms between 12 hour and 72 hour (t-test details of Fig. S4).** The FDR controlling procedure based on bonferroni correction was applied to adjust the type one error.

Gene	P value	Ave 1210	Stdev 1210	Standard Error 1210	Ave 820	Stdev 820	Stdandard Error 820	degree of freedom	FDR adjusted P value	t-statistic	Alpha value
**Camphor**	0.054154058	2.75	1.892969449	0.946484724	4.75	0.957427108	0.478713554	6	0.007766296	-1.885618083	0.05
**Cellulose**	0.017419262	229	71.07742258	35.53871129	378	83.5982456	41.7991228	6	0.058093166	-2.715757561	0.05
**Chitin**	0.025995613	539.5	166.7683023	83.38415117	812.5	152.2136656	76.10683281	6	0.073462196	-2.41818703	0.05
**Cutin**	0.029241137	76.75	30.08183283	15.04091642	121	23.13727152	11.56863576	6	0.0710638	-2.331977355	0.05
**Glyoxylate cycle**	0.045893264	233.5	78.04912983	39.02456491	361.75	101.3619751	50.68098756	6	0.057076724	-2.005013041	0.05
**Hemicellulose**	0.024730023	416.25	130.0163451	65.00817256	641.25	129.2165495	64.60827475	6	0.049354577	-2.454908646	0.05
**Inulin**	0.012963917	5	2.160246899	1.08012345	8.5	1	0.5	6	0.056743087	-2.940588176	0.05
**Lactose**	0.000456841	10	0.816496581	0.40824829	17	2.160246899	1.08012345	6	0.052551298	-6.062177826	0.05
**Lignin**	0.030766418	141.5	43.89760814	21.94880407	218.75	51.04491486	25.52245743	6	0.049102825	-2.294856771	0.05
**Other**	0.028470257	9.5	3.31662479	1.658312395	15.25	3.593976442	1.796988221	6	0.044779196	-2.351513709	0.05
**Pectin**	0.023364801	308.75	97.75948377	48.87974188	466.75	80.37982749	40.18991374	6	0.043999489	-2.496808289	0.05
**Phospholipids**	0.016720894	49.25	13.40087062	6.700435309	70.25	7.365459931	3.682729966	6	0.041424944	-2.746604202	0.05
**protein**	0.016787272	13.5	2.380476143	1.190238071	21.25	5.123475383	2.561737691	6	0.040233008	-2.743613519	0.05
**Starch**	0.026340704	1235.5	364.6500606	182.3250303	1829.5	332.1580949	166.0790474	6	0.046552947	-2.408499587	0.05
**Tannins**	0.00683449	18.25	6.238322424	3.119161212	29.75	2.362907813	1.181453907	6	0.048766462	-3.447845769	0.05
**Terpenes**	0.038337721	48.5	18.85912688	9.42956344	75.25	16.5	8.25	6	0.048761593	-2.135024076	0.05
**Vanillin/Lignin**	0.043029231	85	34.44802849	17.22401424	129.25	25.96632435	12.98316217	6	0.054154058	-2.051539144	0.05

Table S 3. **The detailed results of one-tailed independent t-test on the overall carbon degradation groups in oil/oil with dispersant microcosms between 824m and 1210 m (t-test details of Fig. 8a).** The false discovery rate (FDR) controlling procedure based on Bonferroni correction was applied to adjust the type one error.

Gene	P value	Ave 1210	Stdev 1210	Stdandard Error 1210	Ave 820	Stdev 820	Stdandard Error 820	Degree of freedom	t-statistic	FDR ajusted P value	Alpha
**amyA**	0.026080622	1025	312.4686651	156.2343325	1519.5	264.5064586	132.2532293	6	-2.415788183	0.06018605	0.05
**chitinase**	0.028786912	348	108.8577053	54.42885264	519.25	97.52392869	48.76196434	6	-2.343422703	0.057573825	0.05
**acetylglucosaminidase**	0.019015352	180.5	56.09812831	28.04906416	281.25	51.33143936	25.66571968	6	-2.649958889	0.071307572	0.05
**AceB**	0.043752287	142.5	49.00680225	24.50340112	219.25	57.12194558	28.56097279	6	-2.039503217	0.04687745	0.05
**ara**	0.032588866	145	51.14032981	25.57016491	217	38.34057903	19.17028951	6	-2.252935422	0.048883299	0.05
**xylanase**	0.023309975	132.5	41.58926143	20.79463072	203.5	38.73413654	19.36706827	6	-2.498544468	0.063572659	0.05
**cellobiase**	0.027085237	104.75	39.17801254	19.58900627	175.75	44.73160702	22.36580351	6	-2.388044699	0.058039793	0.05
**phenol_oxidase**	0.036323251	101	29.06314963	14.53157482	152.75	37.70389016	18.85194508	6	-2.174134668	0.047378153	0.05
**AceA**	0.049104656	81.25	29.11328448	14.55664224	130.75	41.39545064	20.69772532	6	-1.956213601	0.05079792	0.05
**endoglucanase**	0.010251282	71.25	22.99818833	11.49909417	126.75	27.09704781	13.5485239	6	-3.123148614	0.076884613	0.05
**cutinase**	0.029241137	76.75	30.08183283	15.04091642	121	23.13727152	11.56863576	6	-2.331977355	0.054827132	0.05
**xyla**	0.043742448	73.75	27.01080031	13.50540015	119.75	36.12363031	18.06181515	6	-2.039665642	0.04860272	0.05
**vana**	0.043315594	71.25	28.2415179	14.12075895	105.75	18.40968948	9.204844739	6	-2.046747981	0.051978713	0.05
**cda**	0.038984596	70.5	19.15724406	9.57862203	103.75	24.78406746	12.39203373	6	-2.122911113	0.048730745	0.05
**mannanase**	0.008601792	63.5	11.09053651	5.545268253	98.25	18.19111505	9.095557524	6	-3.262096017	0.086017924	0.05
**RgaE**	0.01575878	61.5	21.07921567	10.53960784	96	12.90994449	6.454972244	6	-2.79144121	0.067537627	0.05
**pula**	0.031933429	61.25	18.24600413	9.123002064	90	17.60681686	8.803408431	6	-2.267725599	0.050421204	0.05
**glucoamylase**	0.033893017	57	14.89966443	7.449832213	84	19.16594202	9.582971008	6	-2.224401897	0.048418595	0.05
**rgl**	0.043342922	56	15.29705854	7.64852927	78.25	15.45692941	7.728464703	6	-2.046292446	0.050011064	0.05
**pectinase (pectate_lyase)**	0.020021239	51.75	15.34872416	7.67436208	77.75	12.68529332	6.342646661	6	-2.611450197	0.066737465	0.05
**pme**	0.020396144	47.75	13.57387196	6.78693598	72.75	13.64734406	6.823672032	6	-2.597621667	0.061188433	0.05
**exoglucanase**	0.015464186	36	7.874007874	3.937003937	52.5	8.736894948	4.368447474	6	-2.805759996	0.07732093	0.05
**pec_Cdeg**	0.035391094	25.75	10.30776406	5.153882032	39.5	7.141428429	3.570714214	6	-2.192993613	0.048260582	0.05
**limeh**	0.030863152	24.25	8.539125638	4.269562819	38.25	8.732124598	4.366062299	6	-2.292567539	0.051438587	0.05
**glx**	0.024543243	23.25	7.228416147	3.614208074	37	8.524474568	4.262237284	6	-2.460495077	0.061358107	0.05
**cdh**	0.058549831	21	8.286535263	4.143267632	31.25	7.544313532	3.772156766	6	-1.829312314	0.058549831	0.05
**tannase_Cdeg**	0.00683449	18.25	6.238322424	3.119161212	29.75	2.362907813	1.181453907	6	-3.447845769	0.205034703	0.05
**phospholipase_C_fungi**	0.013296099	18.5	6.244997998	3.122498999	28.75	3.201562119	1.600781059	6	-2.92112905	0.079776593	0.05
**phospholipase_D_fungi**	0.007719839	19.25	3.403429643	1.701714821	26	2.160246899	1.08012345	6	-3.348937834	0.115797591	0.05
**nplT**	0.029316245	17	2.160246899	1.08012345	24.75	6.291528696	3.145764348	6	-2.330102687	0.051734551	0.05

Table S 4. The detailed results of one-tailed independent t-test on the most abundant 30 carbon degradation genes in oil/oil with dispersant microcosms between 824m and 1210 m (t-test details of Fig. 8b). The FDR controlling procedure based on Bonferroni correction was applied to adjust the type one error.

Gene	P value	Ave 1210	Standard Error 1210	Ave 820	Standard Error 820	Degree of freedom	Alpha	t-statistic	FDR adjusted P value
**phn**	0.057798842	110.8202779	23.1614243	167.3233295	20.19772897	6	0.05	-1.838628353	0.079331744
**catechol**	0.041545935	102.7925126	20.24169798	162.9109798	20.69225806	6	0.05	-2.076887016	0.078600418
**nagg**	0.041427367	108.9116575	19.92471065	161.8400025	15.84845636	6	0.05	-2.07895282	0.082854735
**tfda**	0.035945226	94.73634191	20.04150256	154.7203409	18.82157174	6	0.05	-2.181721932	0.093191326
**nitroreductase_1**	0.028322367	94.86772378	15.37901056	144.3670128	14.32321262	6	0.05	-2.355324795	0.090116621
**alkb**	0.047661843	90.21049796	16.76706438	137.7332327	17.21209995	6	0.05	-1.977727639	0.077589047
**exaa(moxf)**	0.050038325	89.11979925	18.70204526	137.5001529	16.44604322	6	0.05	-1.942627631	0.076145278
**pcag**	0.054078088	87.45310018	15.83550761	128.2929893	14.75903832	6	0.05	-1.88663063	0.080541834
**poba**	0.057353947	80.98567509	16.74551922	126.3525977	18.02038802	6	0.05	-1.84420396	0.080295526
**arylest**	0.05557344	73.59501361	12.64455967	114.3743704	17.8106343	6	0.05	-1.866955784	0.081044601
**nfsa_2**	0.040779166	78.85670625	8.514981187	110.890671	12.741283	6	0.05	-2.090354057	0.083957106
**atza**	0.023439879	70.32579731	11.81477877	107.3505776	8.984640274	6	0.05	-2.494437729	0.109386103
**bphF1**	0.035176754	65.76980469	10.66189363	103.6114209	13.52365391	6	0.05	-2.197402374	0.098494912
**nitrilase**	0.021331049	62.40823783	11.93194792	102.8630166	10.32081417	6	0.05	-2.564280888	0.114859493
**nhh**	0.027631682	62.71698299	12.50868632	101.7640854	10.68640618	6	0.05	-2.37340334	0.092105607
**one_ring_23diox**	0.036365096	66.93741994	12.760008	101.408823	9.421446868	6	0.05	-2.173299796	0.09091274
**badh**	0.010490557	64.1456403	9.444127934	99.95191701	6.617226064	6	0.05	-3.105040373	0.146867802
**mdla**	0.044352269	62.42870886	15.5098414	98.56717733	8.744529992	6	0.05	-2.029668226	0.079606637
**nfsb_2**	0.059983407	66.79535611	5.374123432	90.7376276	12.07208602	6	0.05	-1.811852156	0.080746894
**mult_ring_12DiOx**	0.046847442	56.9386867	11.18250597	81.63562435	5.380277943	6	0.05	-1.990162891	0.079983438
**catb**	0.047254988	50.90777895	11.16516599	80.30035031	9.738415935	6	0.05	-1.983912973	0.078758314
**dehh109**	0.02008557	46.10330302	7.414214708	79.38502665	10.38030072	6	0.05	-2.60905786	0.127817266
**catechol_b**	0.037504886	41.64675929	9.356817738	71.75330223	10.40986973	6	0.05	-2.150931466	0.082041938
**cmci**	0.036734937	41.21915044	9.24877286	68.77535114	8.736075549	6	0.05	-2.165963633	0.088670539
**nitroreductase_b**	0.025320446	43.13411187	6.348159391	64.51979805	6.05597533	6	0.05	-2.437537248	0.098468402
**one_ring_12diox**	0.044252727	36.56133106	7.049849279	57.15343703	7.284745183	6	0.05	-2.031290561	0.081518182
**tfth**	0.036952496	33.06983784	5.373389836	54.14414937	8.134507512	6	0.05	-2.161683537	0.086222491
**atzb**	0.009703197	27.66535408	5.149496487	48.52929335	4.110852453	6	0.05	-3.166425652	0.169805948
**phta**	0.02400902	26.25496369	5.878390213	42.7688323	3.146707084	6	0.05	-2.476723877	0.105039463
**mauab**	0.060202806	27.26747166	6.290608564	39.59578448	2.619387741	6	0.05	-1.8092164	0.07951314
**atzc**	0.034867563	28.01007507	3.778720879	39.03813074	3.280567388	6	0.05	-2.203811184	0.101697059
**rd**	0.023355305	24.57907596	2.006791882	34.94542112	3.634061568	6	0.05	-2.497108683	0.116776525
**nitro**	0.076944925	24.81439543	3.795702111	34.64001987	4.675381744	6	0.05	-1.63157501	0.091290589
**proO**	0.103532676	22.42603816	4.69209053	33.45055004	6.226130236	6	0.05	-1.414091633	0.113238864
**mhpa**	0.072337465	22.79063079	5.515562471	32.24432571	1.174770432	6	0.05	-1.676400102	0.090421831
**trzn**	0.018704049	17.2366624	3.178549827	28.99581014	3.066868315	6	0.05	-2.662317814	0.163660428
**hbh**	0.038121973	18.33636467	4.088068122	28.88386562	2.756876509	6	0.05	-2.13911067	0.080864791
**bco**	0.008192856	13.69315542	2.041131954	26.05383516	3.139193565	6	0.05	-3.301084488	0.191166635
**nbac**	0.003210221	17.26138783	1.967124289	25.81773726	0.710172432	6	0.05	-4.091221124	0.224715459
**dehh**	0.035436768	14.33404141	4.287660282	25.78991485	2.987959677	6	0.05	-2.192057704	0.095406682
**amie**	0.024241385	14.98222742	2.366891434	25.35274425	3.468634455	6	0.05	-2.469618715	0.099817469
**pcpb**	0.027166217	16.77422704	2.248005081	24.81386895	2.510263103	6	0.05	-2.385855499	0.09508176
**tutfdg**	0.04516646	14.51265608	1.02738182	24.03592425	4.60948306	6	0.05	-2.01653543	0.079041304
**aln**	0.018854722	13.62216726	3.059243403	22.77656774	1.586795026	6	0.05	-2.656308824	0.146647841
**dmsa**	0.026861742	12.29740934	2.794725723	21.93013319	2.894472032	6	0.05	-2.39412234	0.098964312
**bph**	0.013008267	13.65374302	1.745065118	21.17116095	1.871309977	6	0.05	-2.93795918	0.151763111
**p450aro**	0.065485712	13.58282471	2.367700912	20.32562218	3.044039993	6	0.05	-1.748447897	0.083345452
**xamo**	0.15495977	15.26406496	3.298531507	19.55544425	2.023691819	6	0.05	-1.108930235	0.159517411
**oxdb**	0.02123955	9.767512524	2.288003087	19.28604854	2.917106449	6	0.05	-2.567474787	0.123897376
**tfdb**	0.080555387	11.41058515	3.762904765	18.34624324	2.161701541	6	0.05	-1.59821342	0.092440608
**atzd**	0.019032213	7.609126799	1.391601108	15.70647031	2.72123334	6	0.05	-2.649295673	0.133225493
**cpna**	0.031784027	9.172352811	2.477532532	15.53216071	1.305122424	6	0.05	-2.271140975	0.096733994
**trze**	0.074511146	9.946602278	2.179563998	14.70008975	1.870778651	6	0.05	-1.654920624	0.089927245
**pchcf**	0.082123808	10.55005979	1.249795049	14.21279864	1.945200727	6	0.05	-1.584162994	0.092720429
**scnabc**	0.037269089	8.58093985	1.868274947	13.30584407	1.146518927	6	0.05	-2.155501153	0.084156006
**xyll**	0.063132902	6.491140593	0.887616362	9.662742059	1.550881886	6	0.05	-1.774894423	0.081838947
**xlnd**	0.133175641	6.613390348	1.794641349	9.560353788	1.600969309	6	0.05	-1.225368189	0.141246892
**sdsa**	0.00605104	6.417010282	0.68909201	9.259015108	0.408410276	6	0.05	-3.547944448	0.211786402
**cmua**	0.152887043	6.500863679	1.06312725	8.449205314	1.378129275	6	0.05	-1.119389109	0.159732731
**adpb**	0.086094403	5.91166773	1.347182198	8.232367858	0.653951475	6	0.05	-1.54970033	0.095660447
**toma**	0.055732064	5.361314849	1.183656328	7.818336643	0.578607979	6	0.05	-1.86489969	0.079617235
**apc**	0.017522777	4.152978755	0.982520939	7.160090204	0.514552263	6	0.05	-2.711298273	0.175227768
**ophc**	0.041542652	3.713993218	1.177408766	7.159150204	1.168418382	6	0.05	-2.076944142	0.080777378
**assa**	0.079505224	4.015188818	0.95691183	6.426598444	1.154932473	6	0.05	-1.607767464	0.092756095
**cdd**	0.232447301	4.921999668	1.574312832	6.267671494	0.704214539	6	0.05	-0.780262987	0.232447301
**nicdehydr**	0.074211575	3.868148168	0.040093162	6.0091421	1.290809626	6	0.05	-1.657844713	0.091137022
**trza**	0.048600897	3.949822748	0.709999602	5.856958023	0.662695978	6	0.05	-1.963652109	0.077319608
**Dyp**	0.048973759	3.274388764	1.007535365	5.492132024	0.517303782	6	0.05	-1.958139018	0.076181403
**qorl**	0.108310704	3.611726033	0.776962824	4.755137981	0.286733422	6	0.05	-1.38062696	0.116642297
**fcba**	0.185605774	3.376487885	0.851514387	4.532732401	0.84065428	6	0.05	-0.966300248	0.188295713

Table S 5. The detailed results of one-tailed independent t-test on the most abundant 70 organic remediation genes in oil/oil with dispersant microcosms between 824m and 1210 m. The FDR controlling procedure based on Bonferroni correction was applied to adjust the type one error.
